# Cell phones and CHWs: a transformational marriage?

**DOI:** 10.9745/GHSP-D-14-00007

**Published:** 2014-02-11

## Abstract

Mobile phones can be transformative for community health workers (CHWs) in enhancing their influence and status and helping to solve practical problems. While formal intervention research can help advance mHealth application, most progress will come through a “diffusion of innovation” process.

Community health workers (CHWs) have a key role to play if we are to achieve our ambitious goals to reduce child and maternal mortality globally. A wide diversity of CHW cadres have been established in many countries, both in the public sector and with nongovernmental organizations, yet several major challenges have emerged:

- Limited population coverage
- Motivation and retention (especially when CHWs are volunteers)
- Supervision
- Quality of service
- Best constellation of services for the most impact, without overburdening the CHW

While mHealth is no panacea, it can help address each of these challenges substantially, notably via one simple technology—the increasingly ubiquitous cell phone. One of the strengths of CHWs is their presence in, and connection with, their communities. Among other things, this link enables them to promote crucial behavior change interventions. But ironically, being in the community can also isolate them from the rest of the health system. Moreover, despite their key service delivery role, their typically low education, skill level, and social status can limit their influence and effectiveness within the overall health system.

In this issue of GHSP, however, Campbell et al. demonstrate that use of mobile phones, accompanied by provision of good technical content, can markedly strengthen the role that CHWs play in delivering services in Malawi.^1^ Using the Net-Map methodology, which assesses the roles and influences in the health system's social network, the authors find that providing CHWs with a cell phone plus content relevant to their jobs transformed the role of the CHW, from almost a social nonentity to a major hub in the health system's social network. Crawford and colleagues, also in Malawi, find that SMS messaging for pregnant women and caregivers of children under 1, often sent through CHWs as intermediaries, was effective in improving both the knowledge and intended positive behavior of clients.^2^ Lastly, Brunie and colleagues examine what motivates volunteer CHWs in Uganda.^3^ They find that provision of cell phones is itself a motivator. But more importantly, mobile phones can enhance many of the other key motivators: gains in knowledge and skills, social status, and client appreciation as well as ability to ameliorate challenges such as commodity stockouts, emergencies, and even disease outbreaks. So yes, it seems the cell phone can be a transformative technology for the CHW.

But this immense potential for mobile phones raises the question of how best to promote mHealth more generally. Clearly, there is an important place for formal intervention research. However, because situations vary so widely and technology is exploding and changing so rapidly, most progress will likely come through a “diffusion of innovation” model,^4^ where knowledge of successful implementation diffuses rapidly through less formal channels and people try out new approaches and adapt quickly and flexibly to their specific situation.

We must rely on human ingenuity to adapt and apply mHealth in a continuous fashion in each country context. It is already happening spontaneously across the world and that will only accelerate. For example, social media has already become a popular way to share health information with a wide range of audiences and networks; it seems plausible in the future that some social media tools could build on the existing network of CHWs and enhance their connectedness even more. As a global health community, our best role is to facilitate such innovation, by helping to connect the innovators and early adopters and through ongoing assessments of what is and isn't working well. Formal intervention research has a definite role, but it takes considerable time to carry out. Above all, we must not allow it to stand in the way. *– Global Health: Science and Practice*
